# A Sequence-Based Novel Approach for Quality Evaluation of Third-Generation Sequencing Reads

**DOI:** 10.3390/genes10010044

**Published:** 2019-01-14

**Authors:** Wenjing Zhang, Neng Huang, Jiantao Zheng, Xingyu Liao, Jianxin Wang, Hong-Dong Li

**Affiliations:** School of Information Science and Engineering, Central South University, Changsha 410083, China; wjzhang@csu.edu.cn (W.Z.); huangneng@csu.edu.cn (N.H.); jiantao@csu.edu.cn (J.Z.); liaoxingyu@csu.edu.cn (X.L.); jxwang@csu.edu.cn (J.W.)

**Keywords:** genomics, read quality assessment, third-generation sequencing

## Abstract

The advent of third-generation sequencing (TGS) technologies, such as the Pacific Biosciences (PacBio) and Oxford Nanopore machines, provides new possibilities for contig assembly, scaffolding, and high-performance computing in bioinformatics due to its long reads. However, the high error rate and poor quality of TGS reads provide new challenges for accurate genome assembly and long-read alignment. Efficient processing methods are in need to prioritize high-quality reads for improving the results of error correction and assembly. In this study, we proposed a novel Read Quality Evaluation and Selection Tool (REQUEST) for evaluating the quality of third-generation long reads. REQUEST generates training data of high-quality and low-quality reads which are characterized by their nucleotide combinations. A linear regression model was built to score the quality of reads. The method was tested on three datasets of different species. The results showed that the top-scored reads prioritized by REQUEST achieved higher alignment accuracies. The contig assembly results based on the top-scored reads also outperformed conventional approaches that use all reads. REQUEST is able to distinguish high-quality reads from low-quality ones without using reference genomes, making it a promising alternative sequence-quality evaluation method to alignment-based algorithms.

## 1. Introduction

Next-generation sequencing (NGS) dominated the DNA sequencing area since its development, dramatically reducing the cost and error of sequencing by enabling a massively paralleled approach capable of producing large numbers of reads [1]. With the length generated by most NGS machines being short (often less than 200 bp), the applications of NGS are limited in gene/transcript reconstruction and complex genomic assembly [2,3,4].

The emergence of third-generation sequencing (TGS) technology offers many new prospects for genome research, especially thanks to its dramatically increased reads length [5], to solve complex genome regions with long repeats [6,7,8,9]. In 2014, Oxford Nanopore Technologies (ONT) presented their tiny MinION sequencer. The MinION can produce reads thousands of bases long. Scientists used this technology to construct genomes of new species [6], such as vaccinia virus [10], *Saccharomyces cerevisiae* [11], and tobacco [12]. The one-dimensional (1D) reads from MinION have a raw nucleotide accuracy less than 75%, while the two-dimensional (2D) reads are of higher quality (80–88% accuracy) [13].

The standard for judging assembly and long transcripts is mapping rate or genome coverage, which depends on alignment and, therefore, is time-consuming. The accuracy of second-generation sequencing is about 99.96%; however, it still needs to be corrected in assembly, scaffolding, and gap-filling [7,14,15]. At the same time, the genomes of many species are incomplete, leading to the fact that part of reads cannot be aligned to the genome and to the limitation of downstream analysis. There are more widely used alignment methods currently available, such as bowtie [16], HISAT (Hierarchical Indexing for Spliced Alignment of Transcripts) [17], BLAT (BLAST-like alignment tool) [18], and Tophat2 [19]. Currently, there are several assembly algorithms, such as de Bruijn graph (DBG), string graph, and overlap layout consensus (OLC) [20]. The DBG algorithm, which splits the reads into k-mers and builds the overlap graph, is a fast assembler suitable for large-scale SGS reads. 

However, these tools were originally designed for NGS and do not work well for TGS reads. The high error rate of TGS poses new challenges for long-read alignment, assembly, structure variation [21], etc. To solve this problem, some error correction methods were put forward, including hybrid error correction methods, such as LoRDEC (a hybrid error correction method) [22], LSC (a computational method to perform error Correction of TGS Long reads by SGS short reads) [23], proovread [24], and LSCplus [25], which borrow information from high-quality second-generation reads. 

Due to the low quality of data, multiple iterations of error correction are required to achieve assembly quality [26]. Current approaches take all reads as input without filtering, such as MECAT (a fast Mapping, Error Correction, and de novo Assembly Tool) [18], FC_Consensus [27], DAGCon (a Directed Acyclic Graph Consensus method) [28], and FalconSense [29]. The poor-quality reads may have a negative influence on results. MECAT uses different error-correction methods for different types of regions. A counting-based method is used in the regions with consistent matches or deletions without insertion. The local partial order graph (POG) is used in the regions with insertions. The counting-based method greatly improves the calculation speed. The POG method ensures maximum accuracy. The correcting speed of MECAT was about five times higher than that of other tools. The accuracies of MECAT were also consistently higher than those of other two methods.

The alignment tools designed specifically for long reads, such as MECAT, Minimap [30], and BLASR (Basic Local Alignment with Successive Refinement) [31], are still time-consuming for precise alignment. Some tools for long-read processing were also developed. For instance, MECAT is a mapping, error correction, assembly tool, which is very fast compared to several other tools.

Regarding sequencing machines, Pacbio RS, Pacbio RS II, and Nanopore minION are biased toward generating certain types of erroneous nucleotide combinations. For example, an insertion or deletion of the same continuous base was reported in recent studies [11,13]. We assumed that the base content combinations of nucleotides, dinucleotides, and trinucleotides between high-quality and low-quality reads were differential and, therefore, could be used for read-quality evaluation. The nucleotide combinations considered in our work include four kinds of single nucleotide (adenine, A; guanine, G; thymine, T; and cytosine, C), 16 kinds of dinucleotides, and 64 kinds of trinucleotides. Here, the Read Quality Evaluation and Selection Tool (REQUEST) was applied to three real-world third-generation sequencing read datasets from different species. We found that the reads selected by REQUEST were of higher quality and achieved better performances in read correction and contig assembly compared to randomly selected reads. These results support that using high-quality reads rather than all reads is a promising approach for genome assembly.

## 2. Materials and Methods

### 2.1. Data Availability

There are three species of 2D-pass datasets generated by Oxford Nanopore techniques, including *Escherichia coli* (*E. coli*), *Yersinia pestis* (*Yersinia*), and *Drosophila biarmipes* (*Drosophila*). The *E. coli* dataset is available at the Loman lab website (http://lab.loman.net/). The *Yersinia pestis* and *Drosophila biarmipes* datasets are available at the National Center for Biotechnology Information (NCBI) Sequence Read Archive (SRA) database (SRR5117441, SRR7167956). The latest assembled genomes of *E. coli*, *Yersinia*, and *Drosophila* used here were downloaded from the RefSeq database (http://www.ncbi.nlm.nih.gov/refseq).

### 2.2. Methods

**Figure 1:**
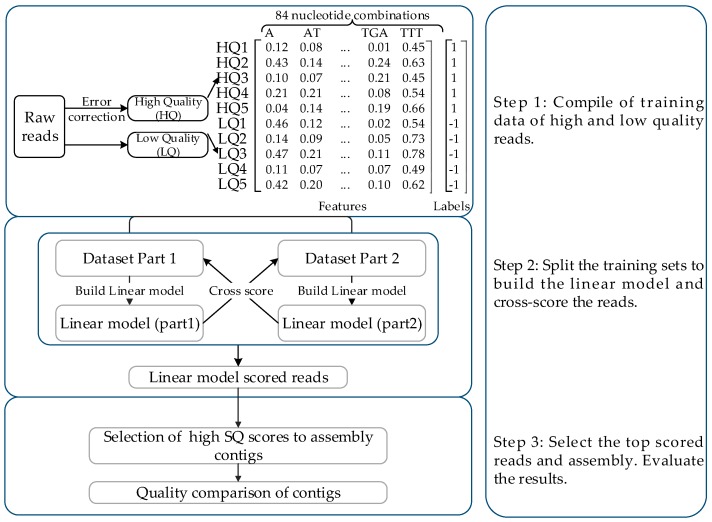
The workflow of the Read Quality Evaluation and Selection Tool (REQUEST). The method consists of three steps: (1) compiling of the training data of high- and low-quality reads; (2) splitting the training set into two parts to build the linear model and cross-score the reads; (3) selecting the top-scored reads and evaluating them. SQ stands for the score of sequencing read quality computed by REQUEST.

REQUEST prioritizes high/low-quality reads based on their differential pattern of nucleotide combinations to evaluate the quality of reads. It consists of three steps to solve the high error rate facing the application of TGS, as shown in Figure 1.

In step 1, to generate the training dataset, the contents of 84 kinds of nucleotide combinations were calculated as the sequence features for each read. The raw reads were regarded as the low-quality reads (LQ, labeled as ‘-1’). The error-corrected reads generated by MECAT with the raw reads as the input data were regarded as the high-quality reads (HQ, labeled as ‘1’).

In step 2, the training sets were split into two subsets to train the linear model separately and cross-score the reads. The process was equivalent to solving a linear least-square problem. The list of predicted Scores of read Quality, denoted as SQ scores, was calculated as shown in Equation (1).

SQ scores = f(X, X_new_) = X_new_ (X^T^X)^−1^ X^T^Y, (1)

where X refers to the matrix of training sets (Part 1 in Figure 1), and X_new_ refers to the matrix of test sets (Part 2 in Figure 1). For all reads, the SQ scores of all raw reads were the combination of SQ scores of the two parts.

In step 3, to verify the effectiveness of the proposed method, we selected the top-ranked 80%, 85%, 90%, and 95% of reads and removed the lowest-scored reads, which reduced the negative impacts. The top-ranked reads could then be used for error correction and contig assembly for testing the effectiveness of our method.

The REQUEST software was implemented in Python and R, and it is freely available at http://github.com/bioinfomaticsCSU/REQUEST.

### 2.3. Evaluation Method

For raw reads and corrected reads, the analytical indicators include the number of reads (Num), as well as the maximum (max), minimum (min), and average length of each dataset. We aligned all reads to the genome and counted the numbers of alignment, aligned rate (%), and mean and median of identity.

Alignments refer to long reads whose overlapped lengths with the reference genome are longer than 2000 bp and where the mismatch rate is less than twice the read error rate [32]. Aligned rate (R) refers to the proportion of reads aligned to genomes in all reads, calculated as

(2) $$\mathrm{aligned}\;\mathrm{rate}=\frac{n}{N}\times 100\%,$$

where n refers to the number of alignments, and N refers to the number of all reads.

The identity is a general standard of sequence quality, showing the degree of match to genome. Identity of a sequence is the ratio of bases aligned to genome, calculated as

(3) $$\mathrm{identity}=\frac{m}{\mathrm{Ref}}\;\times 100\%,$$

where m refers to the number of matched bases, and Ref refers to the length of reference sequence.

We calculated the Pearson correlation coefficient between the identity and SQ scores. The Pearson correlation coefficient was as follows:

(4) $$P=\frac{\sum_{i=1}^{n}\left(Identity_{i}-\bar{Identity}\right)\left(SQ_{i}-\bar{SQ}\right)}{\sqrt{\sum_{i\;=1}^{n}{\left(Identity_{i}-\bar{Identity}\right)}^{2}\sum_{i=1}^{n}{\left(SQ_{i}-\bar{SQ}\right)}^{2}}}.$$

To further investigate whether this selection method could improve assembly, we used the selected datasets for assembly using MECAT2canu with the Nanopore assembly pipeline, and the contigs were evaluated by QUAST (Quality Assessment Tool for Genome Assemblies) [33]. The metrics used here were the number of contigs, max length of contigs, the number of misassemblies (MA), largest alignment, N50, NA50, and genome fraction. N50 is the length of the longest contig such that all the contigs longer than this contig cover at least half of the genome being assembled [34]. NA50 is similar to N50 [35] in corrected contigs. Genome fraction is the percentage of aligned bases in the reference genome. A base in the reference genome is aligned if there is at least one contig covering this base [36].

## 3. Results

### 3.1. High-Quality and Low-Quality Reads Show Different Patterns of Nucleotide Combination Content

**Figure 2:**
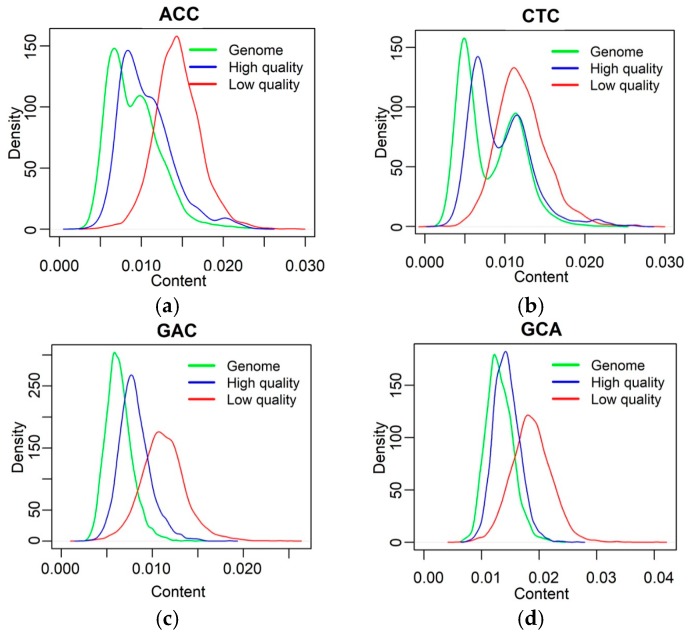
Distribution of nucleotide combinations of genome, high-quality, and low-quality reads of four example trinucleotides: (**a**) ACC; (**b**) CTC; (**c**) GAC; (**d**) GCA. The green, blue, and red lines represent the data from genome (gold-standard error-free reads), high-quality (corrected reads), and low-quality reads (raw reads), respectively.

The differential pattern of long reads was illustrated using a Nanopore sequencing dataset. For instance, Figure 2 shows the difference of four trinucleotides between the reference genome (representing gold-standard error-free reads, green lines), corrected reads (representing high-quality reads, blue lines), and raw reads (representing low-quality reads, red lines). The differences were prominent.

In order to determine whether the selected reads with high SQ scores could result in an improvement of error correction and assembly results, we also randomly selected the same number of raw reads and compared the results between our selected reads and the randomly selected reads. The results of *E. coli* (see Table 1), *Yersinia* (see Table 2), and *Drosophila* (see Table 3) consist of three parts: read alignment, read correction, and contig assembly.

The raw reads were ranked by the SQ scores, and the top 95%, top 90%, top 85%, and top 80% of reads were retained for subsequent analysis. For comparison, subsets of raw datasets of the same size as the reads selected by REQUEST were randomly selected; by repeating this process 20 times, 20 replicate sub-datasets were obtained, and the results on the randomly selected reads were averaged for comparison.

The corrected reads were processed by MECAT. The evaluation criterions of the raw reads and corrected reads contained (1) the number of reads, (2) maximum, minimum, and mean length, (3) the number of alignments and the proportion of alignment in all reads, and (4) mean and median identity.

The evaluation criteria of contigs contained the number of contigs, the maximum contig length, the number of misassemblies, maximum length of alignment, N50, NA50, and genome fraction.

### 3.2. Experimental Results

#### 3.2.1. Results of *Escherichia coli*

**Figure 3:**
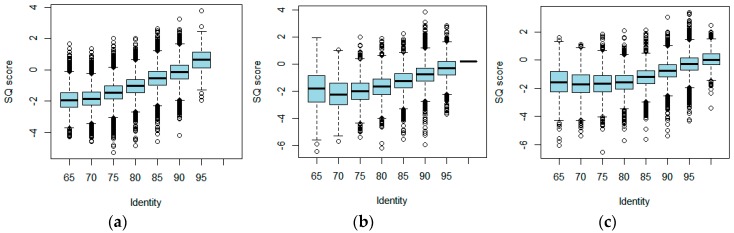
Relationship of identity and predicted (SQ) score. The identity was grouped into 65–70%, 70–75%, 75–80%, 80–85%, 85–90%, 90–95%, and 95–100%. For each group, the distribution of SQ scores was plotted. (**a**) Comparison of *Escherichia coli*; (**b**) comparison of *Yersinia pestis*; (**c**) comparison of *Drosophila biarmipes*.

The raw dataset of *E. coli* contained 31,858 2D reads. The length ranged from 99 bp to 64,218 bp. The identity ranged from 53.95% to 97.42%. We made a comparison between SQ and identity. The relationship between SQ score and identity is shown in Figure 3a. An obvious positive correlation can be seen in the figure. The Pearson correlation coefficient of *E. coli* between identity and SQ score was 0.53 (*p* < 2.2 × 10^−16^), suggesting that the SQ scores are a useful indicator of read alignment-based quality.

Then, error correction and assembly were carried out on the randomly selected reads. The results of *E. coli* datasets are shown in Table 1. The length distribution of the reads selected by our method was higher than that of the randomly selected reads. The proportion of alignments was up to 93.25%, which was 5.57 percent higher than that from randomly selected reads. The distribution of identity had a similar trend. This indicates that SQ scores indeed correlate with the quality of reads.

In the second part, the results of error correction showed different trends. The mean and median identity of the REQUEST selection was lower than that of the random selection in 85–95% and higher in 80%. Meanwhile, the number and length of the REQUEST selection was much higher than random selection. This means that REQUEST allowed more reads to be corrected and the length of effective error correction was longer.

In the last part, the assembly results showed the advantages of REQUEST with fewer and longer contigs. N50 and NA50 were also longer. Although there were slightly more misassemblies, the genome fraction was up to 100%.

#### 3.2.2. Results of *Yersinia pestis*

The raw dataset of *Yersinia* contained 28,429 2D reads. The length ranged from 125 bp to 61,191 bp. The identity ranged from 54.24% to 95.14%. The relationship of SQ score and identity is shown in Figure 3b. The Pearson correlation coefficient of *Yersinia* between identity and SQ score was 0.48 (*p* < 2.2 × 10^−16^). The results are shown in Table 2. The mean length of the reads selected by the REQUEST method was higher than that of the randomly selected reads. The distribution of identity had a similar trend.

In the second part, the results of error correction had similar trends as the *E. coli* datasets. The max length of error-corrected reads was 23,000bp longer than that of random selection.

In the last part, the assembly results also showed the advantages of REQUEST. Overall, the results of model-based selection were comparable to those of all data and outperformed randomly selected reads. The max length, N50, and NA50 were also longer. Although misassemblies were slightly more than the result of random selection, genome fraction was up to 99.96%.

#### 3.2.3. Results of *Drosophila biarmipes*

The raw dataset of *Drosophila* contained 1,375,649 reads. The length ranged from 61 bp to 93,368 bp. The identity ranged from 60.60% to 100.0%. The relationship of SQ score and identity is shown in Figure 3c. The Pearson correlation coefficient of *Drosophila* between identity and SQ score was 0.36 (*p* < 2.2 × 10^−16^). Due to the large genome of *Drosophila*, the results were different from those of the above two datasets (Table 3). The alignment of the REQUEST selected reads was higher than that of all reads and randomly selected reads. In the second part, the number of corrected reads from the REQUEST-selected reads was more than that of randomly selected reads. In the last part, the assembly results also showed the advantage of REQUEST. Overall, the results after selection were better than those without filtering.

## 4. Discussion

In this study, we proposed a sequence-based method, REQUEST, to evaluate and select TGS long reads based on the differential pattern of base combination. It defined the corrected reads as the high-quality reads and the raw reads as the low-quality reads. The base combinations of each read were regarded as the features. REQUEST builds a linear model to score the raw reads. The SQ scores were used as the criterion to select the high-quality reads.

The selected reads with high SQ scores had longer length, higher identity, and higher aligned rate than randomly selected ones. For the results of error correction, the selection generated more reads with longer effective length. The aligned rate of REQUEST was also better than the results of all reads without filtration. Applied to contig assembly, the performance of contigs of REQUEST was better compared to random selection, as well as the performance for all reads in N50, NA50, and other aspects. The genome fraction was higher than that using all reads. It was confirmed that using only reads of high SQ scores had a positive impact in further error correction and assembly. In the future, we plan to test the performance of REQUEST on larger and more complex genomes such as the human genome sequencing data.

REQUEST evaluated and selected third-generation long reads based on the base combinations without a reference genome. It performed better than randomly selected reads and all reads in terms of read quality, error correction, and assembly. REQUEST can quickly evaluate sequence quality, improve the results of error correction and assembly, and reduce the time of iterative error correction of reads generated by the third-generation sequencing technique. REQUEST gives each read an SQ score. In addition to aid filtering low-quality reads, this score can also be integrated with error correction and assembly algorithms for potentially improving their performance.

## Figures and Tables

**Table 1:** Summary of the results of *Escherichia coli* in selection, correction, and contigs. REQUEST—Read Quality Evaluation and Selection Tool.

		P (%)	Num	Max	Min	Mean	n	R (%)	Mean I	Median I
**Read Alignment**	All reads	100	31,858	64,218	99	7668	27,869	87.48	84.16	88.33
	Random	95	30,265	62,072	99	7669	26,471	87.50	84.16	88.33
		90	28,672	62,072	99	7670	25,078	87.50	84.16	88.32
		85	27,079	61,357	100	7670	23,685	87.51	84.15	88.32
		80	25,486	59,926	100	7671	22,288	87.50	84.15	88.32
	REQUEST	95	30,265	64,218	99	7875	27,238	90.00	**84.46**	**88.60**
		90	28,672	64,218	99	7964	26,192	91.35	**84.98**	**89.07**
		85	27,079	64,218	99	8028	25,016	92.38	**85.52**	**89.48**
		80	25,486	64,218	99	8082	23,766	93.25	**86.06**	**89.88**
**Read Correction**	All reads	100	26,034	33,912	2000	8144	25,775	99.01	96.67	98.36
	Random	95	24,252	33,882	2000	8147	24,011	99.01	96.78	98.36
		90	22,943	33,817	2001	8143	22,715	99.01	96.76	98.34
		85	21,626	33,724	2001	8137	21,411	99.00	96.73	98.31
		80	20,303	33,519	2001	8130	20,101	99.00	96.70	98.28
	REQUEST	95	25,715	33,886	2001	8162	25,469	99.04	**96.51**	**98.25**
		90	24,906	33,886	2001	8224	24,670	99.05	**96.59**	**98.29**
		85	23,968	33,880	2001	8279	23,731	99.01	**96.68**	**98.33**
		80	22,883	33,880	2000	8335	22,673	99.08	**96.76**	**98.38**
**Contig Assembly**		**P (%)**	**Num**	**Max(kb)**	**MA**	**Largest alignment (kb)**		**N50 (kb)**	**NA50(kb)**	**GF (%)**
	All reads	100	2	4636	6	2305		4636	1655.60	99.86
	Random	95	3	3724	3	3294		3724	3201.91	99.98
		90	4	2958	3	2606		2947	2438.54	99.97
		85	5	3463	3	3153		3380	3032.37	99.92
		80	7	2496	3	2444		1970	1864.48	99.81
	REQUEST	95	2	4641	5	2530		4641	2529.56	**100.00**
		90	2	4639	7	3587		4639	3587.13	**99.89**
		85	3	4635	5	3956		4635	3956.42	**100.00**
		80	3	4636	5	3957		4636	3956.57	**100.00**

^1^ P indicates the proportion of retained reads; Max, Min, and Mean indicate the maximum, minimum, and mean read lengths, respectively; “n” means the number of alignments; R means the aligned rate; “I” indicates the identity; MA indicates misassemblies; GF indicates genome fraction.

**Table 2:** Summary of the results of *Yersinia pestis* in selection, correction, and contigs.

		P (%)	Num	Max	Min	Mean	n	R (%)	Mean I	Median I
**Read Alignment**	All reads	100	28,429	61,191	125	7679	26,989	94.93	83.44	86.70
	Random	95	27,007	61,191	125	7680	25,628	94.90	83.44	86.70
		90	25,586	61,191	125	7689	24,277	94.88	83.44	86.70
		85	24,164	61,191	145	7679	22,928	94.89	83.44	86.70
		80	22,743	53,492	125	7686	21,573	94.86	83.44	86.70
	REQUEST	95	27,008	61,191	184	7785	26,181	96.94	**83.84**	**86.87**
		90	25,586	61,191	184	7827	25,024	97.80	**84.32**	**87.08**
		85	24,164	61,191	184	7869	23,750	98.29	**84.73**	**87.29**
		80	22,743	61,191	184	7904	22,402	98.50	**85.08**	**87.45**
**Read Correction**	All reads	100	25,776	57,301	2000	7229	24,769	96.09	96.96	98.09
	Random	95	23,953	33,843	2001	7170	23,946	99.97	97.12	98.14
		90	22,633	33,587	2001	7157	22,627	99.97	97.11	98.12
		85	21,315	33,289	2000	7139	21,310	99.98	97.11	98.10
		80	19,974	33,730	2001	7117	19,969	99.98	97.10	98.09
	REQUEST	95	25,357	56,560	2000	7263	25,350	99.97	**96.86**	**98.03**
		90	24,449	56,560	2000	7336	24,442	99.97	**96.93**	**98.07**
		85	23,312	56,587	2000	7399	23,305	99.97	**97.04**	**98.12**
		80	22,028	57,044	2000	7468	22,022	99.97	**97.10**	**98.16**
**Contig Assembly**		**P (%)**	**Num**	**Max(kb)**	**MA**	**Largest alignment (kb)**		**N50(kb)**	**NA50(kb)**	**GF (%)**
	All reads	100	4	4646	30	940		4646	377.69	99.96
	Random	95	5	2749	28	835		2310	370.53	99.72
		90	8	2174	25	816		1642	345.93	99.55
		85	11	1756	28	771		1141	301.66	99.28
		80	19	1194	27	593		471	224.73	98.54
	REQUEST	95	6	4641	31	1012		4641	377.70	**99.96**
		90	4	4658	31	798		4658	377.69	**99.96**
		85	4	4645	29	1012		4645	377.69	**99.96**
		80	7	2571	30	798		2571	282.40	**99.73**

**Table 3:** Summary of the results of *Drosophila biarmipes* in selection, correction, and contigs.

		P (%)	Num	Max	Min	Mean	n	R (%)	Mean I	Median I
**Read Alignment**	All reads	100	1,375,649	93,368	61	4102	845,134	61.44	79.57	82.58
	Random	95	1,306,867	93,368	61	4102	802,968	61.44	79.57	82.24
		90	1,260,870	93,368	61	4101	760,614	60.32	79.57	82.58
		85	1,192,229	93,368	61	4101	718,489	60.26	79.57	82.58
		80	1,123,446	93,368	61	4102	676,352	60.20	79.57	82.58
	REQUEST	95	1,306,867	93,368	83	4298	844,504	64.62	**79.58**	**82.58**
		90	1,260,870	93,368	83	4503	841,439	66.73	**79.61**	**82.61**
		85	1,192,229	93,368	83	4725	833,911	69.95	**79.65**	**82.67**
		80	1,123,446	93,368	105	4950	818,457	72.85	**79.72**	**82.79**
**Read Correction**	All reads	100	628,180	53,163	2000	6743	625,472	99.57	89.25	94.68
	Random	95	594,932	52,702	2000	6654	592,270	99.55	89.22	94.67
		90	571,876	52,531	2000	6579	558,019	97.58	89.21	94.68
		85	536,463	49,260	2000	6452	522,184	97.34	89.19	94.68
		80	498,685	47,746	2000	6297	483,630	96.98	89.19	94.69
	REQUEST	95	634,003	53,154	2000	6713	630,933	99.52	89.10	94.55
		90	633,478	53,154	2000	6715	629,206	99.33	89.11	94.56
		85	632,026	53,154	2000	6719	629,206	99.55	89.14	94.56
		80	627,427	53,157	2000	6731	575,145	91.67	89.25	94.72
**Contig Assembly**		**P (%)**	**Num**	**Max(kb)**	**MA**	**Largest alignment (kb)**		**N50(kb)**	**NA50(kb)**	**GF (%)**
	All reads	100	2185	673	10,602	304		67	31.00	55.65
	Random	95	2051	530	9689	216		57	27.00	46.36
		90	1868	301	8973	176		50	23.00	36.92
		85	1635	226	8165	160		43	16.00	28.09
		80	1385	191	7376	112		39	10.00	20.90
	REQUEST	95	2164	552	10,815	307		68	31.00	**55.82**
		90	2142	552	10,732	234		68	31.00	**55.75**
		85	2132	552	10,616	234		68	31.00	**55.57**
		80	2113	552	10,734	234		67	31.00	**54.95**

## References

[1] Treangen T.J., Salzberg S.L. (2012). Repetitive DNA and next-generation sequencing: computational challenges and solutions. Nat. Rev. Genet..

[2] Alkan C., Sajjadian S., Eichler E.E. (2010). Limitations of next-generation genome sequence assembly. Nat. Methods.

[3] Abnizova I., Leonard S., Skelly T., Brown A., Jackson D., Gourtovaia M., Qi G., Te B.R., Faruque N., Lewis K. (2012). Analysis of context-dependent errors for Illumina sequencing. J. Bioinform. Comput. Biol..

[4] Abnizova I., Skelly T., Naumenko F., Whiteford N., Brown C., Cox T. (2010). Statistical comparison of methods to estimate the error probability in short-read Illumina sequencing. J. Bioinform. Comput. Biol..

[5] Lu H., Giordano F., Ning Z. (2016). Oxford Nanopore MinION Sequencing and Genome Assembly. Genom. Proteom. Bioinform..

[6] Li C., Lin F., An D., Wang W., Huang R. (2018). Genome Sequencing and Assembly by Long Reads in Plants. Genes.

[7] Li M., Tang L., Liao Z., Luo J., Wu F., Pan Y., Wang J. (2018). A novel scaffolding algorithm based on contig error correction and path extension. IEEE/ACM Trans. Comput. Biol. Bioinform..

[8] Li M., Tang L., Wu F., Pan Y., Wang J. (2018). SCOP: A novel scaffolding algorithm based on contig classification and optimization. Bioinformatics.

[9] Liao X., Li M., Luo J., Zou Y., Wu F., Pan Y., Luo F., Wang J. (2018). Improving de novo assembly based on read classification. IEEE/ACM Trans. Comput. Biol. Bioinform..

[10] Prazsák I., Tombácz D., Szűcs A., Dénes B., Snyder M., Boldogkői Z. (2018). Full Genome Sequence of the Western Reserve Strain of Vaccinia Virus Determined by Third-Generation Sequencing. Genome Announc..

[11] Jenjaroenpun P., Wongsurawat T., Pereira R., Patumcharoenpol P., Ussery D.W., Nielsen J., Nookaew I. (2018). Complete genomic and transcriptional landscape analysis using third-generation sequencing: A case study of Saccharomyces cerevisiae CEN.PK113-7D. Nucleic Acids Res..

[12] Lu P., Jin J., Li Z., Cao P., Fan K., Xu Y. (2017). Genome assembly based on the third-generation sequencing technology and its application in tobacco. Tobacco Sci. Technol..

[13] Ip C.L., Loose M., Tyson J.R., De C.M., Brown B.L., Jain M., Leggett R.M., Eccles D.A., Zalunin V., Urban J.M. (2015). MinION Analysis and Reference Consortium: Phase 1 data release and analysis. F1000Research.

[14] Wu B., Wang J., Luo J., Li M., Wu F., Pan Y. (2018). MEC: Misassembly error correction in contigs using a combination of paired-end reads and GC-contents. IEEE/ACM Trans. Comput. Biol. Bioinform..

[15] Li M., Wu B., Yan X., Luo J., Pan Y., Wu F., Wang J. (2017). PECC: Correcting contigs based on paired-end read distribution. Comput. Biol. Chem..

[16] Langmead B., Trapnell C., Pop M., Salzberg S.L. (2009). Ultrafast and memory-efficient alignment of short DNA sequences to the human genome. Genome Biol..

[17] Daehwan K., Ben L., Salzberg S.L. (2015). HISAT: A fast spliced aligner with low memory requirements. Nat. Methods.

[18] Kent W.J. (2002). BLAT—The BLAST-like alignment tool. Genome Res..

[19] Kim D., Pertea G., Trapnell C., Pimentel H., Kelley R., Salzberg S.L. (2013). TopHat2: Accurate alignment of transcriptomes in the presence of insertions, deletions and gene fusions. Genome Biol..

[20] Sović I., Križanović K., Skala K., Šikić M. (2016). Evaluation of hybrid and non-hybrid methods for de novo assembly of nanopore reads. Bioinformatics.

[21] Zhang Z., Wang J., Luo J., Ding X., Zhong J., Wang J., Wu F., Pan Y. (2016). Sprites: Detection of deletions from sequencing data by re-aligning split reads. Bioinformatics.

[22] Leena S., Eric R. (2014). LoRDEC: Accurate and efficient long read error correction. Bioinformatics.

[23] Kin Fai A., Underwood J.G., Lawrence L., Wing Hung W. (2012). Improving PacBio long read accuracy by short read alignment. PLoS ONE.

[24] Hackl T., Hedrich R., Schultz J., Förster F. (2014). proovread: Large-scale high-accuracy PacBio correction through iterative short read consensus. Bioinformatics.

[25] Hu R., Sun G., Sun X. (2016). LSCplus: A fast solution for improving long read accuracy by short read alignment. BMC Bioinform..

[26] Sameith K., Roscito J.G., Hiller M. (2017). Iterative error correction of long sequencing reads maximizes accuracy and improves contig assembly. Brief. Bioinform..

[27] Chin C.S., Peluso P., Sedlazeck F.J., Nattestad M., Concepcion G.T., Clum A., Dunn C., O’Malley R., Figueroabalderas R., Moralescruz A. (2016). Phased diploid genome assembly with single-molecule real-time sequencing. Nat. Methods.

[28] Koren S., Schatz M.C., Walenz B.P., Martin J., Howard J.T., Ganapathy G., Wang Z., Rasko D.A., Mccombie W.R., Jarvis E.D. (2012). Hybrid error correction and de novo assembly of single-molecule sequencing reads. Nat. Biotechnol..

[29] Berlin K., Koren S., Chin C.S., Drake J.P., Landolin J.M., Phillippy A.M. (2015). Assembling large genomes with single-molecule sequencing and locality-sensitive hashing. Nat. Biotechnol..

[30] Li H. (2017). Minimap2: Pairwise alignment for nucleotide sequences. Bioinformatics.

[31] Chaisson M.J., Tesler G. (2012). Mapping single molecule sequencing reads using basic local alignment with successive refinement (BLASR): Application and theory. BMC Bioinform..

[32] Xiao C.L., Chen Y., Xie S.Q., Chen K.N., Wang Y., Han Y., Luo F., Xie Z. (2017). MECAT: Fast mapping, error correction, and de novo assembly for single-molecule sequencing reads. Nat. Methods.

[33] Gurevich A., Saveliev V., Vyahhi N., Tesler G. (2013). QUAST: Quality assessment tool for genome assemblies. Bioinformatics.

[34] Li M., Liao X., He Y., Wang J., Luo J., Pan Y. (2017). ISEA: Iterative Seed-Extension Algorithm for De Novo Assembly Using Paired-End Information and Insert Size Distribution. IEEE/ACM Trans. Comput. Biol. Bioinform..

[35] Luo J., Wang J., Shang J., Luo H., Li M., Wu F.X., Pan Y. (2018). GapReduce: A gap filling algorithm based on partitioned read sets. IEEE/ACM Trans. Comput. Biol. Bioinform..

[36] Luo J., Wang J., Zhang Z., Li M., Wu F.X. (2016). BOSS: A novel scaffolding algorithm based on an optimized scaffold graph. Bioinformatics.

